# *Alkanna tinctoria* leaves extracts: a prospective remedy against multidrug resistant human pathogenic bacteria

**DOI:** 10.1186/s12906-015-0646-z

**Published:** 2015-04-23

**Authors:** Usman Ali Khan, Hazir Rahman, Muhammad Qasim, Anwar Hussain, Azizullah Azizllah, Waheed Murad, Zakir Khan, Muhammad Anees, Muhammad Adnan

**Affiliations:** Department of Microbiology, Kohat University of Science and Technology, Kohat, Pakistan; Department of Botany, Abdul Wali Khan University, Mardan, Pakistan; Department of Botany, Kohat University of Science and Technology, Kohat, Pakistan; Department of Pharmacy, University of Lahore, Punjab, Pakistan

**Keywords:** *Alkanna tinctoria* leaves extracts, Anti-MDR bacterial activity, MICs and MBCs, Phytochemicals analysis

## Abstract

**Background:**

Plants are rich source of chemical compounds that are used to accomplish biological activity. Indigenously crude extracts of plants are widely used as herbal medicine for the treatment of infections by people of different ethnic groups. The present investigation was carried out to evaluate the biological potential of *Alkanna tinctoria* leaves extract from district Charsadda, Pakistan against multidrug resistant human pathogenic bacteria including *Acinetobacter baumannii*, *Escherichia coli*, *Pseudomonas aeruginosa* and *Staphylococcus aureus*.

**Methods:**

Anti-multi-drug resistant bacterial activity of aqueous, chloroform, ethanol and hexane extracts of *Alkanna tinctoria* leaves were evaluated by well diffusion method. Minimum inhibitory concentrations (MICs) and minimum bactericidal concentrations (MBCs) of different extracts were determined. Moreover qualitative phytochemicals screening of the studied extracts was performed.

**Results:**

All four selected bacteria including *A. baumannii*, *E. coli*, *P. aeruginosa* and *S. aureus* were categorized as multi-drug resistant (MDR) as they were found to be resistant to 13, 10, 19 and 22 antibiotics belonging to different groups respectively. All the four extract showed potential activity against *S. aureus as compare to* positive control antibiotic (Imipenem). Similarly among the four extracts of *Alkanna tinctoria* leaves, aqueous extract showed best activity against *A. baumannii* (10 ± 03 mm), *P. aeruginosa* (12 ± 0.5 mm), and *S. aureus* (14 ± 0.5 mm) as compare to Imipenem. The MICs and MBCs results also showed quantitative concentration of plant extracts to inhibit or kill MDR bacteria. When phytochemicals analysis was performed it was observed that aqueous and ethanol extracts showed phytochemicals with large number as well as volume, especially Alkaloides, Flavonoides and Charbohydrates.

**Conclusion:**

The undertaken study demonstrated that all the four extracts of *Alkanna tinctoria* leaves exhibited considerable antibacterial activity against MDR isolates. Finding from the current study will be helpful for further elucidation of lead molecules from *Alkanna tinctoria* leaves for future therapeutic use against MDR pathogens.

## Background

Plants act as reservoirs for wide variety of secondary metabolites including alkaloids, flavonoids, tannins and terpenoids which possess therapeutic properties [1]. The antimicrobial potential of plants have been studied by a large number of researchers across the globe [2,3]. According to World Health Organization (WHO) in 91 countries there are nearly 2000 medicinal plants [4]. Medicinal plants are still being used by rural communities with increasing popularity for treating or preventing various infections. In Pakistan limited data is available on the therapeutic uses of medicinal plants. Among these medicinal plants *Alkanna tinctoria* is under investigation as having several therapeutic uses [5]; however has not been documented for its anti-multi-drug resistant bacterial activity and phytochemicals content.

Microorganisms resistant to more than two groups of antibiotics are regarded as MDR [6]. Recent emergence of antibiotic resistance trigger research to explore medicinal plants for their antimicrobial protection against MDR pathogens because there is need to discover new antibiotic therapies to combat resistance to older drugs [7,8]. Hospital acquired infections caused by MDR bacteria is major challenge for clinician [4].

The present explored anti-MDR bacterial activity of *Alkanna tinctoria* leaves from district Charsadda, Pakistan. Moreover, phytochemicals analysis of the *Alkanna tinctoria* leaves was also performed. Finding from the present study will be helpful for elucidation of bioactive molecules from *Alkanna tinctoria* leaves which can be used to treat infections caused by MDR bacterial pathogens.

## Methods

The undertaken study was accomplished to check the antibacterial activity and phytochemicals content of *Alkanna tinctoria* leaves against MDR clinical bacterial isolates. The already characterized clinical isolates on the bases of 16rRNA gene sequencing including *A. baumannii and E. coli* were taken from patients having urinary tract infections while *P. aeruginosa* and *S. aureus* were isolated from patients having wound infections admitted in the tertiary care hospital of Peshawar. The isolates were reconfirmed on the basis of culture, microscopy and biochemical characteristics. An informed written consent was taken from patient to use the samples for the current study. Ethical approval was granted by the Departmental Research Ethics Committee of Kohat University of Science and Technology (DREC KUST).

### Antibiotic susceptibility pattern of clinical isolates

The susceptibility of the clinical isolates to commonly used antibiotics was determined on Mueller-Hinton agar (MHA) using disc diffusion method [9]. The results were interpreted as resistant (R), intermediate sensitive (I) and sensitive (S) as described by Clinical and Laboratory Standard Institute (CLSI formerly NCCLS) guidelines 2007 [10].

### Collection and processing of *Alkanna tinctoria*

The *Alkanan tinctoria* leaves were collected from Charsadda region of Pakistan and identified at the Herbarium of Botany Department, KUST, where the voucher specimen was deposited under reference number 10050/ATL. The grinding process was performed as described [11]. *Alkanna tinctoria* leaves were first washed, dried and then chopped into small pieces. After slicing the plant was dried and mashed into powder. After the plant grinding process, 1000 g of dried plant powder was soaked in 4 liter of each selected solvent (aqueous, chloroform, ethanol and hexane) and kept it for 21 day. T she plant extracts were then filtered with a sterilized filter paper. The filtrate plant’s extracts were then processed in rotary evaporator at 125 rpm on 44°. After evaporation solidified extracts were kept in sterile small air tight glass bottles. Then 250 mg of each extract was added in 10 ml DMSO achieving the concentration of 25 mg/ml. The tubes were kept on rotary shaker at 125 rpm for 1 hour to obtain homogeneous mixture [12].

### Anti-MDR bacterial activity of *Alkanna tinctoria* leaves

Activity of *Alkanna tinctoria* leaves were evaluated against selected MDR bacteria by using well diffusion method [9]. In each plate four extracts (aqueous, chloroform, ethanol and hexane) of *Alkanna tintoria* leaves were poured in each well separately. DMSO was incorporated as negative control. For positive control a standard antibiotic disc (Imipenem) was applied on the medium in the centre of the plate. All the plates were sealed and incubated at 37°C for 24 hours. Results were measured by noted zone of inhibition in millimeters (mm) around the wells and disc [13].

### Determination of minimum inhibitory concentrations (MICs) and minimum bactericidal concentrations (MBCs)

Briefly, the stock extracts (100 mg/ml) were prepared in nutrient broth and serially diluted as 50 mg/ml, 25 mg/ml, 12.5 mg/ml and 6.25 mg/ml. Then 100 μl of overnight bacterial broth cultures were matched to 0.5 McFarland standard and inoculated in all the tubes. One tube was inoculated with nutrient broth and bacterial culture but without extract as positive control while tubes having nutrient broth and extract but without bacterial culture was incorporated as negative control. The tubes were incubated at 37°C for 24 hours. The lowest concentration at which no growth appeared was considered as MIC.

MBC was processed with subsequent steps of MICs. Each selected MIC’s tubes content were inoculated on prepared nutrient agar plates. The plates were incubated at 37°C for 18 hours. The lowest concentration at which no growth appeared was considered as MBC of specific plant against selected bacteria [14,15].

### Phytochemicals screening

Phytochemicals analysis of secondary metabolites such as Alkaloids, Bufadienoloides, Charbohydrate, Flavonoids, Proteins, Resins, Saponins, Tannins, Phenolics, Triterpenoides, Steroids, Gallotannins and Pseudotannins was carried out according to the common phytochemical methods as described by Trease and Evans [16].

### Data analysis

All the experiments were repeated 3 times. Experimental data was reported as mean ± standard error.

## Results

Antibiotic susceptibility of pathogenic bacterial isolates was determined. *A. baumannii* showed resistance to about thirteen antibiotics widely used against this pathogen while *E. coli* was resistant to about ten antibiotics. When *P. aeruginosa* susceptibility pattern was checked it showed resistant to almost nineteen antibiotics while *S. aureus* was resistant to twenty two antibiotics including Vancomycin (Table 1).

**Table 1:** **Evaluation of multi-drug resistant bacterial isolates**

**Antibiotics**	**Bacteria (zone diameter nearest whole mm)**											
	***A. baumannii***			***E. coli***			***P. aeruginosa***			***S. aureus***		
	**R**	**I**	**S**	**R**	**I**	**S**	**R**	**I**	**S**	**R**	**I**	**S**
**Cell wall synthesis inhibitors**												
Amoxicillin	0	-	-	0	-	-	0	-	-	0	-	-
Amoxycillin/Clavulanic acid	-	15 ± 1	-	0	-	-	7 ± 0	-	-	6.5 ± 0	-	-
Ampicillin	7 ± 0.28	-	-	0	-	-	0	-	-	0	-	-
Aztreonam	7 ± 0.11	-	-	0	-	-	0	-	-	0	-	-
Cefepime	-	-	24 ± 0.5	15 ± 0.5	-	-	-	15 ± 0.3	-	-	15 ± 0.5	-
Cefixime	-	-	20 ± 0.5	-	-	19 ± 0.7	-	18 ± 0.3	-	10 ± 0	-	-
Cefotaxime	7 ± 0	-	-	14 ± 0.5	-	-	0	-	-	7 ± 0	-	-
Ceftazidime	-	19 ± 0.7	-	14 ± 0.5	-	-	7 ± 03	-	-	-	15 ± 0.3	-
Ceftriaxone	12 ± 0.5	-	-	-	16 ± 0.5	-	7 ± 0	-	-	10 ± 0.3	-	-
Cephradine (only for *S. aureus*)	NA	NA	NA	NA	NA	NA	NA	NA	NA	6.5 ± 0	-	-
Fosfomycin	-	-	22 ± 0.5	-	-	24 ± 0.5	-	-	30 ± 1	-	-	-
Imipenem	10 ± 0.5	-	-	-	-	23 ± 0.7	10 ± 0	-	-	-	15 ± 0.7	-
Meropenem	10 ± 0.5	-	-	8 ± 0	-	-	7 ± 0	-	-	7 ± 0	-	-
Oxacillin (only for *S. aureus*)	NA	NA	NA	NA	NA	NA	NA	NA	NA	0	-	-
Pipracillin/Tazobactam	-	-	27 ± 1	-	-	18 ± 1	14 ± 0.5	-	-	14 ± 0.3	-	-
Vancomycin (only for *S. aureus*)	NA	NA	NA	NA	NA	NA	NA	NA	NA	9 ± 0.3	-	-
**Cell membrane inhibitors**												
Colistin sulphate	0	-	-	-	-	12 ± 0.3	-	-	11 ± 0.3	10 ± 0	-	-
**Protein synthesis inhibitors**												
Amikacin	-	15 ± 0.3	-	-	15 ± 0.5	-	9	-	-	7 ± 0	-	-
Chloramphenicol	NA	NA	NA	NA	NA	NA	12 ± 0.5	-	-	9 ± 0	-	-
Doxycycline	-	13 ± 0.5	-	-	-	14 ± 0.5	7 ± 0.3	-	-	10 ± 0.3	-	-
Erythromycin	0	-	-	7 ± 0	-	-	7 ± 0	-	-	8 ± 0	-	-
Gentamycin	9 ± 0.28	-	-	-	-	15 ± 0.5	9 ± 0	-	-	10 ± 0.3	-	-
Tetracycline	7 ± 0	-	-	-	-	18 ± 0.3	0	-	-	7 ± 0	-	-
Tigecyclin	-	-	19 ± 0.7	-	18 ± 0.5	-	-	15 ± 0.5	-	-	15 ± 0.5	-
**DNA inhibitors**												
Ciprofloxacin	15 ± 0.5	-	-	-	-	22 ± 0.5	12 ± 0.5	-	-	11 ± 0.3	-	-
Nitrofurantoin	-	15 ± 0.5	-	8 ± 0.3	-	-	0	-	-	8 ± 0	-	-
**Folate pathway inhibitors**												
Trimethoprim/Sulphamethoxazole	0	-	-	-	11 ± 0	-	0	-	-	0	-	-

± = standard error of given value, – = not observed, NA = not applicable R = resistant, I = intermediate sensitive and S = sensitive.

After confirmation of MDR pathogens, aqueous, chloroform, ethanol and hexane extracts of *Alkanna tinctoria* leaves were evaluated *in vitro* against *A. baumannii*, *E. coli*, *P. aeruginosa* and *S. aureus*. The concentration of all plant extracts were set to 25 mg/ml. Average zone of inhibition were recorded for all experiments performed three times. It was observed that *Alkanna tinctoria* showed maximum medicinal potential against *S. aureus* as compared to other three bacteria. Chloroform and ethanolic extracts showed highest zone of inhibition (19 mm) and aqueous extract showed 14 mm inhibitory zone against *S. aureus*. Zone of inhibition of the aqueous, chloroform, ethanol and hexane extracts of *Alkanna tinctoria* leaves against *A. baumannii*, *E. coli*, *P. aeruginosa* appeared in the range of 9 mm to 14 mm as shown in Table 2.

**Table 2:** **Antibacterial activity of** ***Alkanna tinctoria*** **(leaves) against MDR pathogens**

**MDR bacteria**	**Aqueous extract (mm)**	**Chloroform extract (mm)**	**Ethanol extract (mm)**	**Hexane extract (mm)**	**+Ve IPM (mm)**	**-Ve DMSO (mm)**
*A. baumannii*	10 ± 0.3	9 ± 0.3	11 ± 0.5	10 ± 0.3	10 ± 0.5	0
*E. coli*	11 ± 0.5	14 ± 0.5	14 ± 0.5	13 ± 0.3	23 ± 0.7	0
*P. aeruginosa*	12 ± 0.5	9 ± 0.3	11 ± 0.3	10 ± 0.3	10 ± 0	0
*S. aureus*	14 ± 0.5	19 ± 0.7	19 ± 0.5	15 ± 0.5	15 ± 0.7	0

± = standard error of given value, +Ve IPM = Imipenem as positive control and -Ve DMSO = Dimethyl sulfoxide as negative control.

The MICs were calculated to check the bacteriostatic effect of plant extracts. The range of MICs of selected four plant extracts against selected MDR isolates were in between 6.25 mg/ml to 25 mg/ml (Table 3).

**Table 3:** **Minimum inhibitory concentration (MICs) and minimum bactericidal concentration (MBCs) of** ***Alkanna tinctoria*** **(leaves) against MDR isolates**

**MDR bacteria**	**Aqueous extract (mg/ml)**		**Chloroform extract (mg/ml)**		**Ethanol extract (mg/ml)**		**Hexane extract (mg/ml)**	
	**MICs**	**MBCs**	**MICs**	**MBCs**	**MICs**	**MBCs**	**MICs**	**MBCs**
*A.baumannii*	12.5	>100	25	>100	25	>100	25	>100
*E. coli*	25	>100	25	>100	25	>100	25	>100
*P.aeruginosa*	25	>100	25	>100	12.5	>100	25	>100
*S. aureus*	12.5	>100	12.5	>100	12.5	>100	25	>100

>100 shows concentration greater than 100, + = no bacterial growth observed and - = bacterial growth observed.

Aqueous extract inhibited *A. baumannii* and *S. aureus* at minimum concentration of 12.5 mg/ml, while *E. coli* and *P. aeruginosa* were inhibited at lower concentration of 25 mg/ml. MIC of hexane extract against all four MDR bacteria were recorded as 25 mg/ml.

The MBCs were calculated to check the bactericidal effect of plant extracts against MDR pathogens. MBCs of most of the extracts the plant found to be higher than 100 mg/ml which was the maximum concentration used for the experiment (Table 3).

After checking the qualitative and quantitative measurement of antibacterial activity of *Alkanna tinctoria* leaves the phytochemicals contents were analyzed. Alkaloids were detected in all four extracts of *Alkanna tinctoria* leaves but aqueous and ethanol extracts contained in higher amount than chloroform and hexane extracts which showed less amount. Bufadienoloides and Resins were present in moderate quantity. Flavonoides and Gallotannins were only present in hexane and aqueous extracts respectively. Carbohydrates, Phenolics and Triterpenoides were not detected in any of the four extracts. Proteins and Tannins were found to be in lower concentration. Steroides and Pseudotannins were appeared in higher and moderate volumes except ethanol and aqueous extracts which lower to undetectable amount respectively. Saponins of all extracts of this plant were in lower volume (Table 4).

**Table 4:** **Phytochemicals composition of** ***Alkanna tinctoria*** **(leaves)**

**Phytochemicals**	**Aqueous extract**	**Chloroform extract**	**Ethanol extract**	**Hexane extract**
Alkaloides	+++	+	+++	+
Bufadienoloides	+	++	++	++
Charbohydrates	_	_	_	_
Flavonoides	_	_	_	+++
Gallotannins	+++	_	_	_
Phenolics	_	_	_	_
Proteins	+	+	+	+
Pseudotannins	_	++	++	++
Resins	+	++	++	++
Saponins	+	+	+	+
Steroides	+++	+++	+	+++
Tannins	_	+	+	+
Triterpenoides	_	_	_	_

- sign shows not detected, + shows compound present in small amount; ++ shows compound present in moderate amount; +++ shows compound present in higher amount.

## Discussion

Medicinal plants are the rich source of traditional medicines and have been used to treat infectious diseases. The universality and efficacy of medicinal plants is evident from their continued use [17]. *Alkanna tinctoria* leaves is a commonly used as plant herbal therapy for treating different infections including its frequent traditional use in Charsadda, Pakistan.

In the study *Alkanna tinctoria* leaves were selected for the investigation of its qualitative and quantitative potential of antibacterial activity, and further phytochemicals analysis. The aqueous, chloroform, ethanol and hexane extracts were screened against MDR bacteria including *A. baumannii*, *E. coli*, *P. aeruginosa* and *S. aureus*. Tchana et al., (2014) performed similar type of study in Cameroon, in which it was confirmed that medicinal plants had the ability to encountered MDR bacteria [18].

It was observed that *Alkanna tinctoria* leaves extracts showed convincing zone of inhibition against selected MDR pathogens. *S. aureus* appeared to be more sensitive than other three bacteria, this can be the key of previous work of Ghosh and Gaba on *Alkanna tinctoria* wound healing ability [19]. Quick wound healing can be due to the inhibition of *S. aureus* an important pathogen in wound infections. Aqueous, chloroform and ethanolic extracts showed highest zone of inhibition against *S. aureus*, and moderate zone of inhibition against *A. baumannii*, *E. coli and P. aeruginosa*. Unlikely hexane extract did not exhibit as much antibacterial activity against all MDR pathogens. *Alkanna tinctoria* was previously reported as red dye as well as antibacterial [20,21]. As for as literature mining is concerned, the antibacterial activity of *Alkanna tinctoria* leaves extracts of aqueous, chloroform, ethanol and hexane against MDR bacterial isolates were not observed till now in the research literature, as well as phytochemicals analysis of *Alkanna tinctoria* leaves is described here for the first time. Moreover bacteriostatic and bacteriocidal activity *Alkanna tinctoria* leaves was calculated for the first time against MDR pathogens.

By comparing results of inhibitory zone, bacteriostatic concentration and bactericidal concentration of all four extracts of *Alkanna tinctoria* leaves it was obvious that aqueous and ethanol extracts has potential activity against selected MDR pathogens. When phytochemicals analysis was performed it was observed that aqueous and ethanol extracts showed phytochemicals with large number as well as concentration, especially alkaloides, flavonoides and charbohydrates. This shows good solibility of phytochemicals in tested solvents. In phytochemicals results it was observed that the extracts which showed large number of phytochemicals exhibited potential antibacterial activity. One interesting observation we found that the antibacterial activity of aqueous extract against *P. aeruginosa*, and chloroform and ethanolic extracts against *S. aureus* showed better activity as compared to broad spectrum antibiotic Imipenem.

## Conclusion

The current study demonstrated that *Alkanna tinctoria* leaves extracts were efficient against MDR isolates. Phytochemicals analysis showed that *Alkanna tinctoria* leaves have versatile biochemical molecules which may inhibit or kill drug resistant human pathogenic bacteria. Further in-depth analysis will be helpful for future elucidation of bioactive molecules to treat infectious diseases caused by resistant bacteria.

## References

[1] Talib WH, Mahasneh AM (2010). Antimicrobial, cytotoxicity and phytochemical screening of Jordanian plants used in traditional medicine. Molecules.

[2] Morton JF, Swaain T, Kleiman R (1980). Search for carcinogenic principles. Recent advances in phytochemistry.

[3] Anesini E, Perez C (1993). Screening of plants used in Argentine folk medicine for antimicrobial activity. J Ethnopharmacol.

[4] World Health Organization (2002). Use of antimicrobials outside human medicine and resultant antimicrobial resistance in humans. Fact sheet N° 268.

[5] Elsharkawy E, Elshathely M, Jaleel GA, Al-Johar HI (2013). Anti-inflammatory effects of medicinal plants mixture used by Bedouin people in Saudi Arabia. Herba Pol.

[6] Zainuddin ZF, Dale JW (1990). Does Mycobacterium tuberculosis have plasmids?. Tubercle.

[7] D’Costa VM, King CE, Kalan L, Morar M, Sung WW (2011). Antibiotic resistance is ancient. Nature.

[8] Donadio S, Maffioli S, Monciardini P, Sosio M, Jabes D (2010). Antibiotic discovery in the twenty-first century: current trends and future perspectives. J Antibiot.

[9] Kirby WM, Yoshihara GM, Sundsted KS, Warren JH (1956). Clinical usefulness of a single disc method for antibiotic sensitivity testing. Antibiot Annu.

[10] Clinical Laboratory Standards Institute (CLSI) (2007). Performance standards for antimicrobial susceptibility testing; 16th informational supplement. CLSI M100-S16.

[11] Wendakoon C, Calderon P, Gagnon D (2012). Evaluation of selected medicinal plants extracted in different ethanol concentrations for antibacterial activity against human pathogens. J Med Active Plants.

[12] Odey MO, Iwara IA, Udiba UU, Johnson JT, Inekwe UV, Asenye ME (2012). Preparation of plant extracts from indigenous medicinal plants. Int J Sci Tech.

[13] Valgas C, de-Souza SM, Smania EFA, Smânia A (2007). Screening methods to determine antibacterial activity of natural products. Braz J Microbiol.

[14] Kowser MM, Fatema N (2009). Determination of MIC and MBC of selected Azithromycin capsule commercially available in Bangladesh. The ORION Med J.

[15] Petrus EM, Tinakumari S, Chai LC, Ubong A, Tunung R, Elexson N (2011). A study on the minimum inhibitory concentration and minimum bactericidal concentration of Nano Colloidal Silver on food-borne pathogens. Int Food Res J.

[16] Trease GE, Evans WC (1983). Textbook of pharmacognosy.

[17] Bakht J, Khan S, Shafi M (2013). Antimicrobial potentials of fresh *Allium cepa* against gram positive and gram negative bacteria and fungi. Pak J Botany.

[18] Tchana MES, Fankam AG, Mbaveng AT, Nkwengoua ET, Seukep JA, Tchouani FK (2014). Activities of selected medicinal plants against multi-drug resistant gram negative bacteria in Cameroon. Afri Health Sci.

[19] Ghosh PK, Gaba A (2013). Phyto-extracts in wound healing. J Pharm Pharmaceut Sci.

[20] Kreuzner C (2013). *Alkanna tinctoria* (L.) Tausch as purple dye in the recipes of Papyrus Holmiensis and Papyrus Leidensis X. e-Preser Sci.

[21] Genova E, Kitanov G, Stafanova I (1995). Study on the alkannin (shikonin) content in the roots of dyer’s alkanet (*Alkanna tinctoria* (L.). Pharmacia.

